# Characteristics of SARS-CoV-2-specific cytotoxic T cells revealed by single-cell immune profiling of longitudinal COVID-19 blood samples

**DOI:** 10.1038/s41392-020-00425-y

**Published:** 2020-12-04

**Authors:** Qing Xiong, Cheng Peng, Xiaomin Yan, Xueqi Yan, Lin Chen, Beicheng Sun, Shiping Jiao

**Affiliations:** 1 Department of Hepatobiliary Surgery, Nanjing Drum Tower Hospital, The Affiliated Hospital of Nanjing University Medical School, Nanjing, 210093 China; 2 Drum Tower Research Institute of Clinical Medicine, Nanjing University, Nanjing, 210093 China; 3 Department of Infectious Disease, Nanjing Drum Tower Hospital, The Affiliated Hospital of Nanjing University Medical School, Nanjing, 210093 China

**Keywords:** Adaptive immunity, Lymphocytes

**Dear Editor,**

The SARS-CoV-2 virus outbreak that started in December 2019 is now a global pandemic with over 42.7 million confirmed cases and 1.15 million deaths as of October 25, 2020. It has been predicted that resurgence in contagion may be possible as late as 2024.^1^ A persistent anti-SARS-CoV-2 adaptive immune surveillance post infection is critical to lower the risk of resurgence and transmission. T cells are the primary effectors of cellular adaptive immunity that not only clear virus-infected cells during infection but also provide long-term protection post infection as memory cells in some pathogen infections. However, little is known regarding the longitudinal T cell response during and after SARS-CoV-2 infection. Single-cell T cell receptor (TCR) sequencing allows rapid identification of expanded T cell clones that are potentially the antigen-specific clonotypes.^2^ To further characterize the anti-SARS-CoV-2 T cells, we sequenced the circulating T cells of five moderate or severe COVID-19 patients at different stages of infection (two patients at 1, 7 and 16 weeks and three patients at 1 and 17 weeks post symptom onset) and of two healthy donors (Supplementary Tables S1, S2 and S7). T cells with both TCR sequencing and gene expression information from five patients and two health donors were grouped into 16 clusters (Fig. 1a and Supplementary Fig. S1). Naive T cells, naive-effector transitioned T cells, Treg and effector T cells were annotated based on the top 20 differentially expressed genes (DEGs) of each cluster (Fig. 1c and Supplementary Table S3). Effector T cells (Cluster 0, 4, 10, 13, 6, 9 and 14) were characterized on the basis of cytotoxic granules (GZMA/B/K/M/H, PRF1 and GNLY), chemokines (CCL4 and CCL5) and activation markers (NKG7 and FGFBP2) (Fig. 1a, c). Furthermore, the integrated T cell clonality analysis indicated significant clonal expansion in response to SARS-CoV-2 infection (Fig. 1b), whereas no dominant T cell clones were detected in healthy donors (Fig. 1d). The majority of the clonal T cells were localized in the effector T cell area (Fig. 1b). In contrast, only minimal B cell clonal expansion was observed (PA0130–34) (Supplementary Fig. S2). To demonstrate the persistence of the T cell clones, we mapped their clonality at the different time points. The clones identified during the active phase of infection (1 week post symptom onset) showed decreased clonality rate during the course of post infection but persisted till 16 and 17 weeks post symptom onset (Fig. 1d). In addition, the dominant clone in each patient remained so even after 16 and 17 weeks (PA0130–34: 3.53, 2.63, 1.2, 3.35 and 9.17% at timepoint 3, respectively).

**Fig. 1:**
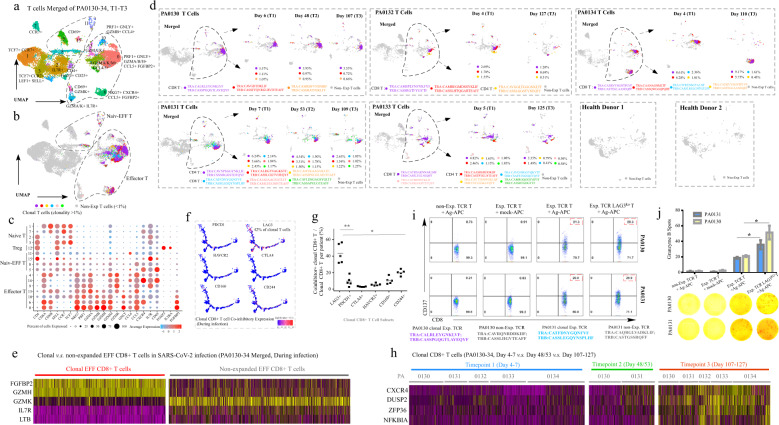
SARS-CoV-2-specific clonal T cells showed signs of long-term survival fate in circulation. **a** Clustering of 22,452 T cells from five patients (PA0130–34, Supplementary Table S7) at three or two consecutive time points and two healthy donors. All T cells are with both TCR and gene expression information. The cell subsets are color-coded. The labeled markers of each cluster in the UMAP are selected from the top 20 differentially expressed genes (DEGs) of each cluster. **b** T cell clones indicated by different colors. Clonal T cells were defined as those T cells with identical CDR3 seq/total T cells ≥1%. Non-clonal T cells are defined as those with the rate <1%. The dashed circles indicate the naive-EFF T and effector T cell population, respectively. **c** Expression of the selected DEGs in each cluster. **d** T cell clonality in five patients at the indicated time points and two healthy donors; the proportion and CDR3 amino acid sequences of the TRA and TRB of each clone across time points is shown. T cell clones are indicated by different colors. **e** DEGs between clonal effector (EFF) CD8+ T cells and non-expanded EFF CD8+ T cells (one cell per clone) measured by the Wilcoxon Rank Sum test. DEGs were identified based on fold change >2 and adjusted *p*-value < 0.01. The analyzed T cells are from an effector T cell population of five patients during virus infection (Timepoint 1). **f** Expression levels of six co-inhibitory molecules in the clonal CD8+ T cells; LAG3 was the dominant co-inhibitory molecule expressed by 42% of clonal T cells. **g** The percentages of clonal CD8+ T cells with the indicated co-inhibitory receptor expression in the entire clonal CD8+ T cell population for each patient. *P < 0.05, ** *P* < 0.01 according to paired *t*-test. **h** DEGs across three consecutive time points for the clonal CD8+ T cells of five patients. Wilcoxon Rank Sum test was used for differential expression analysis; DEGs were selected based on fold change >2 and adjusted *p*-value < 0.01 in two phases. **i** Flow cytometric analysis of TCR-transfected (TCR sequences from clonal T cells or non-expanded T cells) CD8+ T cells co-cultured for 48 h with Ag-APC (virus antigen loading antigen-presenting cell) or mock APC. **j** Representative ELISpot wells from patients PA0130 and 0131 showing granzyme B released by TCR-T cells in response to virus antigens. The experiments were technically repeated three times. The difference between groups was analyzed by unpaired *t*-test

To next identify the distinct cytotoxic characteristics of virus-reactive T cells, we analyzed the DEGs in clonal effector CD8+ T cells relative to those of non-expanded cells of the effector CD8+ T cell population (Cluster 0, 4, 10, 13, 6, 9 and 14) during the active infection phase. Clonal T cells showed significantly higher expression of FGFBP2 and GZMH, which features the active anti-virus cytotoxic T cells (fold change > 2, adjusted *p*-value = 9.94E−160 and 8.94E−129, respectively, Fig. 1e). In contrast, GZMK expression levels were higher in non-expanded effector T cells (fold change > 2, adjusted *p*-value = 5.49E−87). T cell dysfunction is common during viral infections and is driven by induction of co-inhibitory receptors on the cytotoxic effector T cells. The dominant coinhibitory pathway depends on the specific virus.^3^ For instance, PDCD1 or TIM3-overexpressing T cells are the dominant dysfunction population during HIV, HBV, HCV, SIV and LCMV infections. To determine the coinhibitory receptor signature during SARS-CoV-2 infection, we profiled PDCD1, LAG3, CTLA4, HAVCR2 (TIM3), CD160 and CD244 expression in clonal cytotoxic T cells during active infection, and found that the LAG3+ population was dominant (42% of clonal T cells) (Fig. 1f, g). To validate the finding, we analyzed an independent single-cell gene expression and TCR seq dataset (GSE145926) of the bronchoalveolar lavage fluid (BALF) samples of nine COVID-19 patients. Consistently, LAG3+ T cells were dominant (77.36%) in BALF clonal T cells, and 25.23% of clonal T cells were LAG3+ CASP3+ (Supplementary Fig. S3).

Further comparison of the transcriptomes of clonal CD8+ T cells at the different timepoints showed that CXCR4, DUSP2, ZFP36 and NFKBIA were downregulated and upregulated in the clonal CD8+ T cells at timepoints 1 and 3, respectively (fold change > 2, adjusted *p*-value < 0.0001 at both timepoints; Fig. 1h and Supplementary Table S4). CXCR4 is critical for the self-renewal of CD8+ memory T cells, NKFBIA (IK-Bα) promotes transition of terminal effector T cells to memory precursors during virus infection by downregulating NF-κB, and ZFP36 restrains T cell activation and expansion during virus infections. There is little evidence implicating DUSP2 in the cell fate transition of terminal cytotoxic effector T cells, but DUSP2 is known to attenuate signal transducer and activator of transcription 3 and mitogen activated protein kinase signaling. Thus, upregulation of these genes in clonal T cells at 16–17 weeks is highly suggestive of a long-lived memory T cell subset specific for SARS-CoV-2.

To further verify whether the dominant T cell clones in COVID-19 patients are SARS-CoV-2 specific, we ectopically expressed the TCR sequences from the clonal and non-expanded T cells in the autologous T cells (Supplementary Fig. S4a). Briefly, the naive CD8+ T cells were isolated from the blood of PA0130 and PA0131 at timepoint 2 (days 48 and 53 post symptom onset, respectively) (Supplementary Fig. S4b) and transfected with full-length TCRα and β chain mRNA of the selected clones (Supplementary Fig. S4c and Supplementary Table S5). The constant region from murine TCR was inserted to reduce mispairing with endogenous TCR chains and determine transfection efficacy (Supplementary Fig. S4d). As shown in Supplementary Fig. S4d, transgenic TCR β chain expression in the transfected CD8+ T cells was high (at ~80%). We also generated LAG3-knockout T cell clones by co-electroporating LAG3-specific Cas9-sgRNA RNP along with patient-derived TCR mRNA in the TCR transgenic CD8+ T cells (Supplementary Fig. S4e). To generate SARS-CoV-2 antigen-presenting cells (APCs), CD3− immune cells were negatively isolated with CD3 microbeads from the blood of PA0130 and PA0131 (HLA typing in Supplementary Table S6), and transfected with the SARS-CoV-2 structural proteins S, M and N. The TCR transgenic T cells were co-cultured with SARS-CoV-2 Ag-loaded APCs, and the amount of secreted Granzyme B and surface expression of CD137 were measured by ELISpot assay and flow cytometry, respectively (Supplementary Fig. S4a). T cells expressing the expanded clone TCRs released high levels of Granzyme B and generated a CD137+ subset in the presence of SARS-CoV-2 Ag-APCs, while T cells with non-expanded clone TCR were non-responsive (Fig. 1i, j). Furthermore, knocking out LAG3 in the SARS-CoV-2 reactive T cells with expanded clone TCRs not only augmented Granzyme B production and the percentage of CD137+ CD8+ T cells (Fig. 1j, i) but also reduced their apoptosis rates (Supplementary Fig. S4f). Taken together, our data suggest that the dominant expanded T cell clones in COVID-19 patients are likely SARS-CoV-2 reactive.

Longitudinal serological studies of COVID-19 patients have shown that IgG levels declined rapidly in the early convalescent phase (8 weeks post discharge) by 71.1 and 76.2% in the asymptomatic and symptomatic patients, respectively.^4^ Our study also found a limited B cell clonal expansion during the infection course and in the convalescent phase. Although humoral immunity was not lasting, there was evidence showing that T cellular immunity may be lasting and provide long-term protection from resurgence. A recent study demonstrated that blood collected from COVID-19 patients 3–5 weeks post symptom onset harbored T cells reactive to the predicted SARS-COV-2 epitope pool.^5^ Based on our findings, we hypothesize that SARS-CoV-2 infection can induce virus-reactive cytotoxic T cells that persist for more than 17 weeks and possess features of long-term fate in circulation, and may therefore provide lasting cell-mediated immunity against SARS-CoV-2.

## Supplementary information

Supplementary Materials

## Data Availability

The raw sequencing data will be deposited to Genome Sequencing Archive of the National Genomics Data Center shortly; we will provide the accession number when it is available. All other data and materials used in this work are available from the lead corresponding author (jiaoshp@tcrximmune.cn) upon request.
