# Supplementary material for: Characteristics of SARS-CoV-2-specific cytotoxic T cells revealed by single-cell immune profiling of longitudinal COVID-19 blood samples
Source: Signal Transduct Target Ther. 2020 Dec 4;5:285. doi: 10.1038/s41392-020-00425-y (PMC7716113; doi:10.1038/s41392-020-00425-y)
Supplement: Supplementary file 1 — Supplementary Materials [file 41392_2020_425_MOESM1_ESM.pdf]

Supplementary Materials for

Characteristics of SARS-CoV-2 Specific Cytotoxic T Cells Revealed by Single-cell Immune Profiling of Longitudinal COVID-19 Blood Samples

Qing Xiong[1,2 #], Cheng Peng[1,2 #], Xiaomin Yan[3], Xueqi Yan[1,2],

Lin Chen[1,2], Beicheng Sun[1,2 *], and Shiping Jiao[1,2 *]

Correspondence to: sunbc@nju.edu.cn (B.S.); jiaoshp@tcrximmune.cn (S.J.)

This PDF file includes:

Materials and Methods
Fig. S1
Fig. S2
Fig. S3
Fig. S4
Table S1
Table S2
Table S3
Table S4
Table S5
Table S6
Table S7

Materials and Methods

Ethics Statement

The study was conducted in accordance with the Declaration of Helsinki. Ethical approval was obtained from the Research Ethics Committee of Nanjing Drum Tower Hospital. The ethical approval ID is 2020-015-01. All subjects gave their informed consent for sample collection and subsequent analyses.

Subjects and Clinical Sample Collection

Five COVID-19 patients and two healthy donors were recruited from Nanjing Drum Tower Hospital and blood samples were collected from January to June, 2020. The demographic characteristics of the study population were listed in Table 1. All patients had cough and/or fever as the first symptom. SARS-CoV-2 was diagnosed on the basis of clinical symptoms, chest radiography, and sputum and throat swab viral PCR assays. The possibilities of influenza A/B virus, respiratory syncytial virus and adenovirus infection were excluded at the time of enrollment.

Flow Cytometry Analysis

Samples were analyzed using BD Accuri C6 Plus or LSR Fortessa. Cell sorting was performed using a BD FACS Aria III sorter. Cell surface staining for flow cytometry and cell sorting was performed by pelleting and resuspending in 25 μL of FACS buffer (2% FBS in PBS) with antibodies diluted accordingly for 20 min at RT in the dark. The following antibodies were used: PerCP anti-human CD8 (SK1, Biolegend, 309804), FITC anti-human CD197 (CCR7) (G043H7, Biolegend, 353215), APC anti-human CD45RO (UCHL1, Biolegend, 304210) PE anti-human CD45RA (HI100, Biolegend, 304107) FITC anti-mouse TCR β chain (H57-597, Biolegend, 109205) APC anti-human CD3 (HIT3a, Biolegend, 300311). Cells were washed twice in FACS buffer before resuspension and analysis.

Cell Culture

Peripheral blood mononuclear cells (PBMCs) were prepared with Ficoll-Hypaque density gradient centrifugation using human lymphocyte isolation buffer. CD3 negative immune cells were isolated from PBMCs with CD3 Microbeads by negative selection. The purified monocytes were incubated in AIM V medium (Gibco, 12055083). The sorted naïve CD8+ cells were cultured in 10% FBS-RPMI 1640 medium plus 50 U/ml interleukin (IL)-2 (PeproTech, 200-02).

Cell Sorting and Enrichment from PBMC

Naïve CD8+ T cells were purified from PBMC by FACS based on a CD8+ CCR7+ CD45RA+ CD45RO- phenotype. Enrichment of CD3- monocyte cells from PBMC were negative selected by CD3 MicroBeads (Miltenyi Biotec, 130-050-101) following the manufacturer instruction.

HLA Typing

HLA typing of PA0130 and PA0131 were performed with GenDx NGSgo AmpX HLA-A, B, C, DRB1, DQB1(#2841102) and AmpX HLA-DPA1, DPB1, DQA1, DRB3/4/5 (#2841502) following instructions.

TCR Transfection of Naïve CD8+ T cells

The full-length TCR α and β chains were codon optimized for human cell expression system and cloned into the RNA expression vector pGEM-4Z individually. The TCR α and β chains mRNAs were generated by using mMESSAGE mMACHINE (Ambion, AM134555). The naïve CD8+ T-cells stimulated with

human T-activation anti-CD3/28 beads (Dynabeads, Gibco, 11131D) at a cell to beads ratio of 2: 1 were cultured in 12-well plates with 10% FBS-RPMI 1640 medium plus 200 U/ml IL-2 for 48–72 hr. The stimulated naïve CD8+ T-cells were electroporated with mRNA using a 4D-Nucleofector system (Lonza) with a P3 Primary Cell 4D-Nucleofector X Kit S (Lonza, V4XP-3032), 2 million T cells mixed with 3 μg mRNA （1.5 μg α and 1.5 μg β chains） and electroporated, the expression of introduced TCRs were tested overnight.

Antigen Transfection of APC

Patient-derived CD3- immune cells (antigen presenting cells, APC) were loaded with SARS-CoV-2 antigens via electroporation with 2 μg plasmids per $1x10^6$ cells. Three SARS-CoV-2 structural proteins Spike (S), Membrane (M) and Nucleocapsid (N) were synthesized by GenScript, codon optimized and cloned into a mammalian expression vector pUC57, and transfected to antigen presenting cells by electroporation using a 4D-Nucleofector. The APC were co-cultured with T cells 24h after transfection.

Knock Out LAG3 via CRISPR-Cas9

Three LAG3 sgRNAs were synthesized by GenScript (GenCRISPR/Cas9 EasyEdit sgRNA), 50 pmol 2NLS-Cas9 protein (NEB, M0646T) were mixed with 75 pmol sgRNA (three guides mixed, 25 pmol each) then incubated at RT for 15 min to form a RNP solution. The following gRNAs were used: sgRNA 1, ACCUCAGCCCCUGGCUGGAGGUUUUAGAGCUAGAAAUAGCAAGUUAAAAUAAGGCUAGUCCGUUAUCAACUUGAAAAAGUGGCACCGAGUCGGUGCUUUU, sgRNA 2, CUAGUGAAGCCUCUCCAGCCGUUUUAGAGCUAGAAAUAGCAAGUUAAAAUAAGGCUAGUCCGUUAUCAACUUGAAAAAGUGGCACCGAGUCGGUGCUUUU, sgRNA 3, ACCACCGGGACCUCAGCCCCGUUUUAGAGCUAGAAAUAGCAAGUUAAAAUAAGGCUAGUCCGUUAUCAACUUGAAAAAGUGGCACCGAGUCGGUGCUUUU. For LAG3 knock out, primary CD8+ T cells were prepared and cultured as described above. After stimulation for 72h, naïve CD8+ T cells were electroporated with TCR mRNA and RNP using a 4D-Nucleofector with pulse code EH115.

Enzyme Linked Immunosorbent Spot (ELISpot)

For the Granzyme B ELISpot assays, a total of 1×10^4 CD8+ TCR-T cells (16h after TCR transfection) cultured with 2×10^4 APC cells were incubated at 37°C by using a Human Granzyme B ELISpot Kit (BD, 552572). The plates were evaluated using an ELISpot reader ImmunoSpot S6 VERSA, and developed according to manufacturer's instructions after culturing for 48 hr.

Cell Apoptosis Assay

TCR transgenic T cells were co-cultured with Ag loaded monocytes as a ratio of 1:3 for 3 days, the apoptosis assay was performed every 24 hours, apoptotic cells were stained with Annexin V and 7-AAD (BD, 559763) and analyzed by FACS.

Library Preparation for 10X Genomics Single-cell 5’ Gene Expression and V(D)J Sequencing

Single RNA-seq and V(D)J libraries were generated using the 10X Genomics Chromium Controller Instrument and Chromium single cell 5’ library & gel bead kit, along with the V(D)J enrichment kit according to manufacturers’ instructions. Briefly, 8000 peripheral blood mononuclear cells (> 90% viability) were loaded on the controller to generate single-cell Gel Bead-In-Emulsions (GEMs). Reverse transcription and sample indexing were used to generate barcoded cDNA, followed by purification with DynaBeads and PCR amplification. The amplified barcoded cDNA was used to construct 5’ gene expression libraries, TCR and BCR enriched libraries. For 5’ library construction, the amplified cDNA was

fragmented, end repaired, A-tailed, sample indexed and double-sized selected with SPRI beads (average size, 450bp). For the V(D)J library, human T cell and B cell V(D)J sequences were enriched from the amplified cDNA followed by fragmentation, end repairing, A-tailing, sample indexing and double-sized selection with SPRI beads (average size, 600bp). The DNA quantification and fragment size distribution of the libraries were determined with Qubit dsDNA HS assay kit (Thermo, Q32851) and Agilent 2100 BioAnalyzer High Sensitivity DNA kit (Agilent Technologies, 5067-4626). Pooled libraries were then sequenced on an Illumina high output sequencing platform, with 26bp on the first read and 98bp on the second read for gene expression analysis, and 150bp on the two reads for V(D)J libraries.

10X Library Sequencing

The scRNA libraries were sequenced on an Illumina Novaseq to a minimum sequencing depth of 50,000 reads per cell using reads lengths of 26bp read 1, 8bp i7 index, 151bp read 2. The single-cell TCR and BCR libraries were sequenced an Illumina Novaseq to a minimum sequencing depth of 5,000 reads per cell using reads lengths of 151bp read 1, 8bp i7 index, 151bp read 2. For scRNA-seq, we obtained 100,718 cells and a total number of 5,045,836,269 reads with an average of 360,416,876 reads per sample and 50,099 reads per cell, and on average, 19,315 genes were detected per sample. For scTCR-seq, we obtained 42,691 cells and a total number of 467,195,870 reads with an average of 33,371,134 reads per sample and 10,944 reads per cell. For scBCR-seq, we obtained 14,149 cells and a total number of 426,566,323 reads with an average of 30,469,023 reads per sample and 30,148 reads per cell. The single cell mapping statistics are shown in Table S7.

Analyses of scRNA-seq Data

The scRNA-seq reads of each sample were independently aligned to the GRCh38 reference genome (10X Genomics, version 3.0.0) and quantified using cellranger count pipeline (10X Genomics, version 3.1.0) with default parameters. Filtered count matrix of features generated from cellranger count were then analyzed using Seurat (version 3.1.5) [1]. Genes expressed in less than 3 cells and cells with less than/equal to 200 or more than/equal to 5000 expressed genes or cells with more than/equal to 10% mitochondrial counts were discarded. We used the method developed by Yost et al [2] to remove batch effects between samples and cells related to S and G2/M cell cycle phases, a heat-shock gene expression signature, number of UMIs per cell, and mitochondrial RNA content when scaling the data. Variable genes were identified based on average of expression > 0.1 and dispersion > 1, and we used top 5000 variable genes for our analysis. To avoid clustering based on variable V(D)J transcripts, we removed variable TCR and immunoglobulin genes from the list of variable genes. We also removed genes in the S and G2/M cell cycle signature and heat-shock signature to prevent clustering affected by these two factors. Cells were clustered using a shared nearest neighbor (SNN) modularity optimization-based clustering algorithm with the first 50 principal components and resolution set to 0.5 or 3. Clusters were annotated manually based on the expression of known marker genes as indicated.

Analyses of scTCR-seq and scBCR-seq

The reads of scTCR-seq and scBCR-seq were independently aligned to the GRCh38 reference genome (10X Genomics, version 3.1.0) for each sample, and the clonotype analysis and TCR/BCR annotation were carried out using cellranger VDJ pipeline (10X Genomics, version 3.1.0). The percentage of clonotypes was calculated as previously described [3].

Trajectory Analysis

Trajectory analysis was performed by Monocle (version 2.14.0) [4]. Top 5000 highly variable genes were chosen for the differential expression test between clonally expanded CD8+ T cells using differential

GeneTest function, and we used significant genes with q-value< 0.05 as the ordering genes. We removed the effects of 5 covariates including size factor, percentage of mitochondrial counts, expression of the heat-shock gene signature, expression of S and G2/M cell cycle genes in the differential expression test. Dimension reduction was performed using DDRTree.

Quantification and Statistical Analysis

Differential expression analysis in Fig. 1e and Fig. 1h were performed by FindAllMarkers function in the Seurat package, and the test statistic employed was Wilcoxon Rank Sum test. The significant differential genes were defined by fold change > 2 and adjusted *p*-value < 0.01. Paired *t*-test was conducted to assess statistical significance for Fig. 1g, Supplementary Fig. S3d, and Fig. S3f. Unpaired *t*-test was performed for Fig. 1j.

References

1. Stuart, T. et al. Comprehensive Integration of Single-Cell Data. *Cell* **177**, 1888-1902.e1821 (2019).
2. Yost, K.E. et al. Clonal replacement of tumor-specific T cells following PD-1 blockade. *Nat Med* **25**, 1251-1259 (2019).
3. Jiao, S. et al. Differences in Tumor Microenvironment Dictate T Helper Lineage Polarization and Response to Immune Checkpoint Therapy. *Cell* **179**, 1177-1190 e1113 (2019).
4. Trapnell, C. et al. The dynamics and regulators of cell fate decisions are revealed by pseudotemporal ordering of single cells. *Nature biotechnology* **32**, 381-386 (2014).

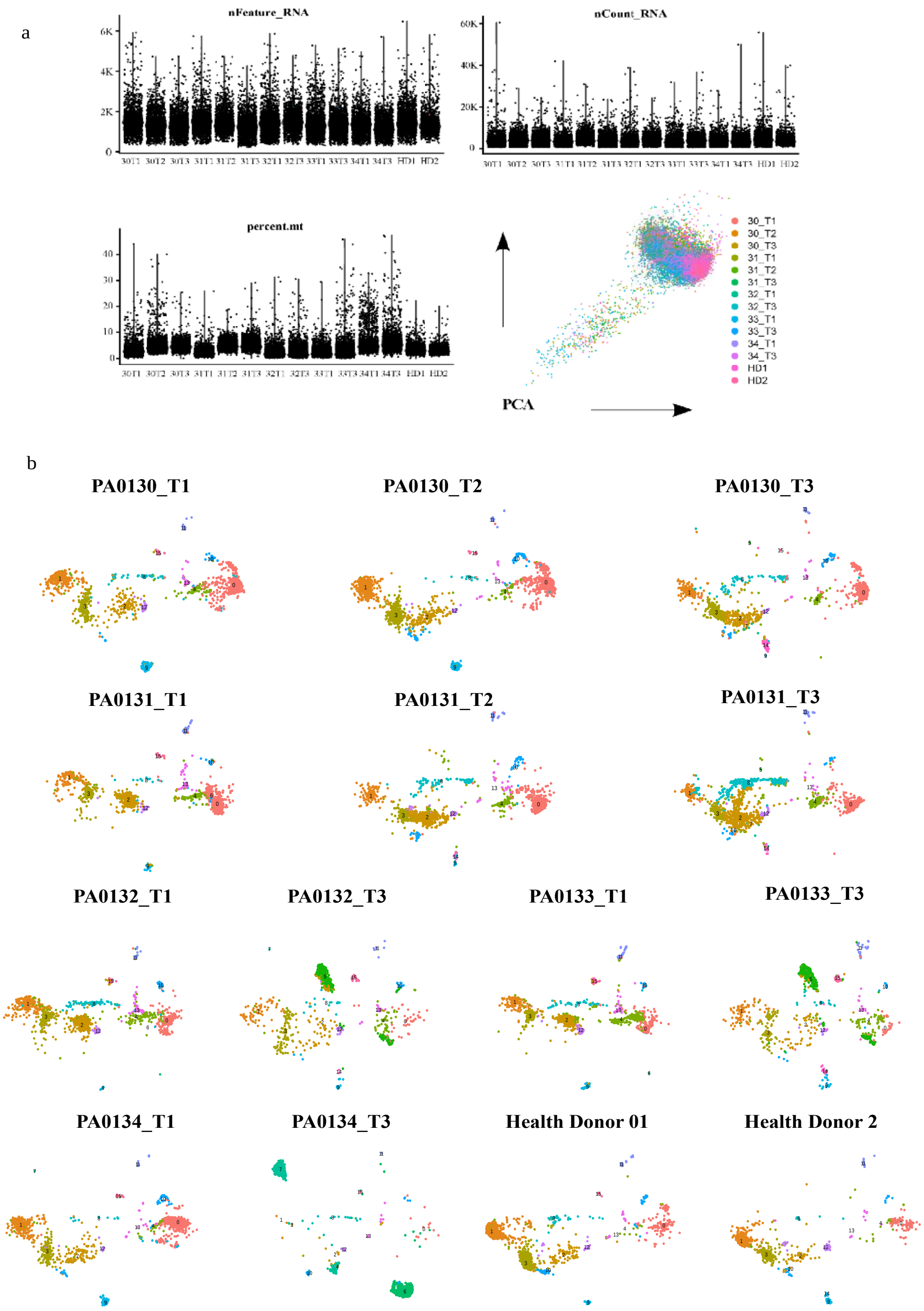

Fig. S1 QC and UMAP of T cells of 5 patients PA0130-34 at 3 consecutive time points and 2 healthy donors. (a) QC and PCA plots of merged T cells of 5 patients PA0130-34 and 2 healthy donors at all timepoints. nFeature_RNA: feature counts; nCount_RNA: read counts; percent.mt: percentage of mitochondrial counts. (b) UMAP of T cell clusters for each sample.

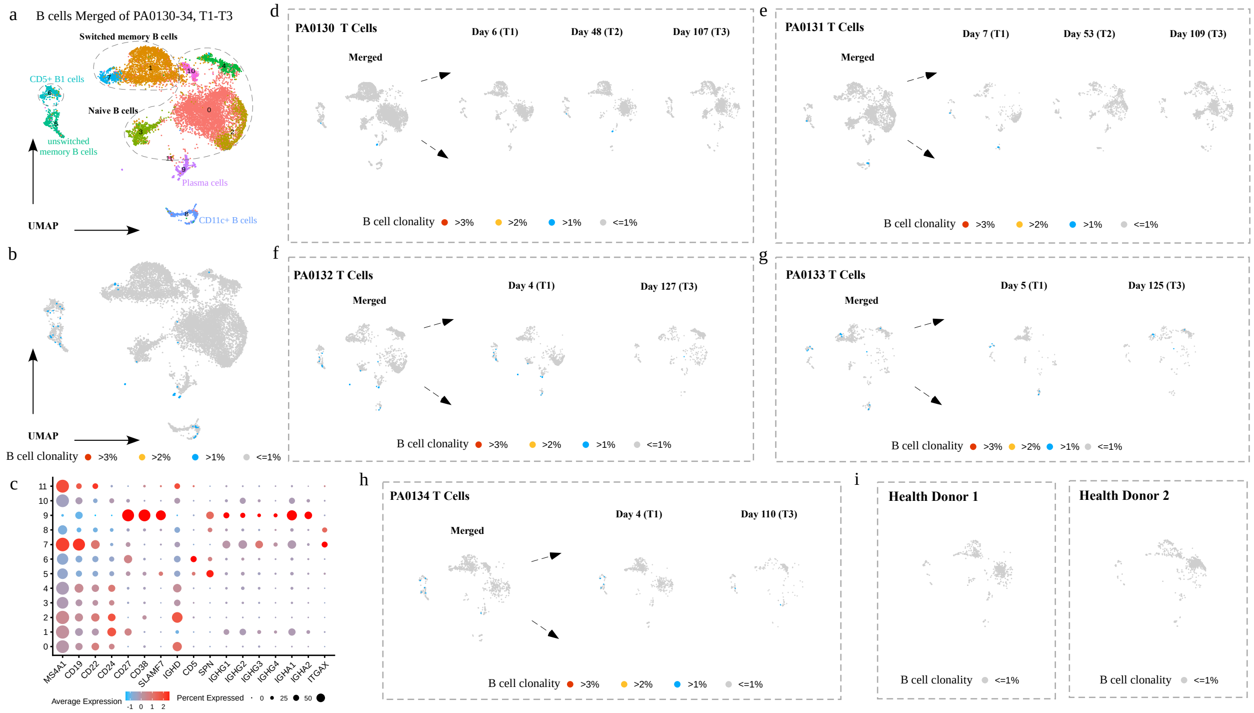

Fig. S2 B cell clonality at consecutive timepoints in each patient and healthy donor. (a) Cell subset clustering of B cells from 5 patients (PA0130-34) at 3 or 2 time points and 2 healthy donors. All B cells are with both BCR and gene expression information. Different colors indicate different cell subsets. (b) Clonal B cells are labeled with colors. (Clonal B cells: B cells with identical CDR3 seq/ total B cells >=1%; Non-clonal B cells with the rate < 1%). Different colors indicate different clones. (c) The expression of the selected core markers in each cluster. (d)-(i) B cell clonality in 5 patients at the indicated time points and two healthy donors; the proportion of each clone across time points was shown.

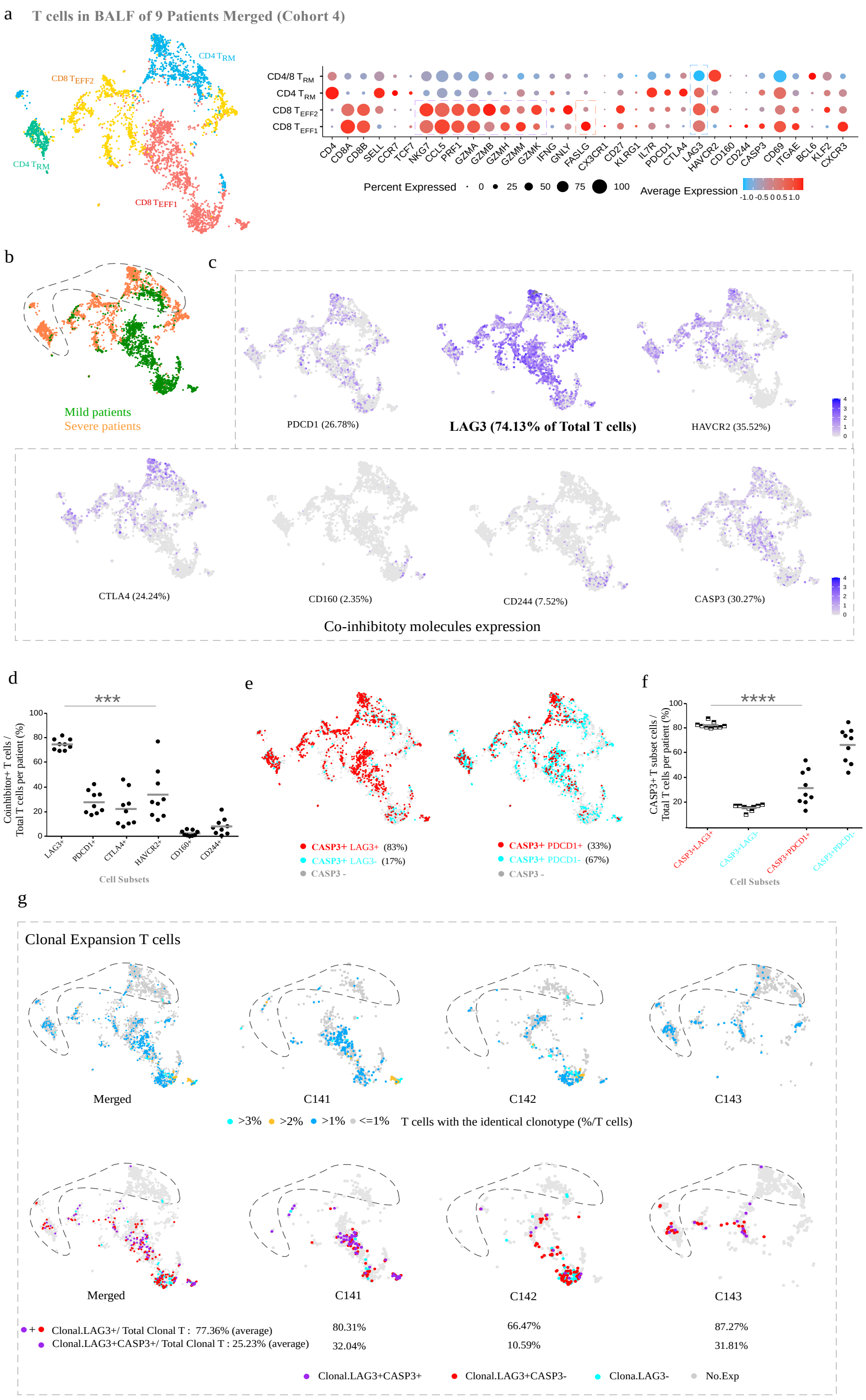

Fig. S3 Large proportion of cytotoxic T cells in the BALF of COVID-19 patients are LAG3+ and apoptotic. (a) Clusters of T cells in BALF samples from 9 COVID-19 patients (3 mild and 6 severe) in GSE145926, and expressions of core markers in each cluster. (b) BALF T cells from patients with mild (Green) and severe disease (Orange). (c) Expression patterns of 6 co-inhibitory molecules in BALF T cells. Proportion of LAG3+ T cells in total BALF T cells was indicated. (d) The percentages of co-inhibitor+ T cell subsets in total BALF T cells for each patient. The difference between groups was examined by paired *t*-test. (e) CASP3+ LAG3+ or CASP3+ PDCD1+ cells were colored as red, and CASP3+ LAG3- or CASP3+ PD1- T cells were colored as blue in the UMAP. (f) The percentages of the 4 subsets in each patient (n=9). CASP3+ LAG3+ cell population is more dominant than CASP3+ PDCD1+ (p value< 0.0001, paired *t*-test). (g) Upper panel: Clonotype analysis of expanded T cells from 3 BALF samples with sufficient cell numbers. Lower panel: Proportion of CASP3+, CASP3+ LAG3+ population in clonally expanded T cells.

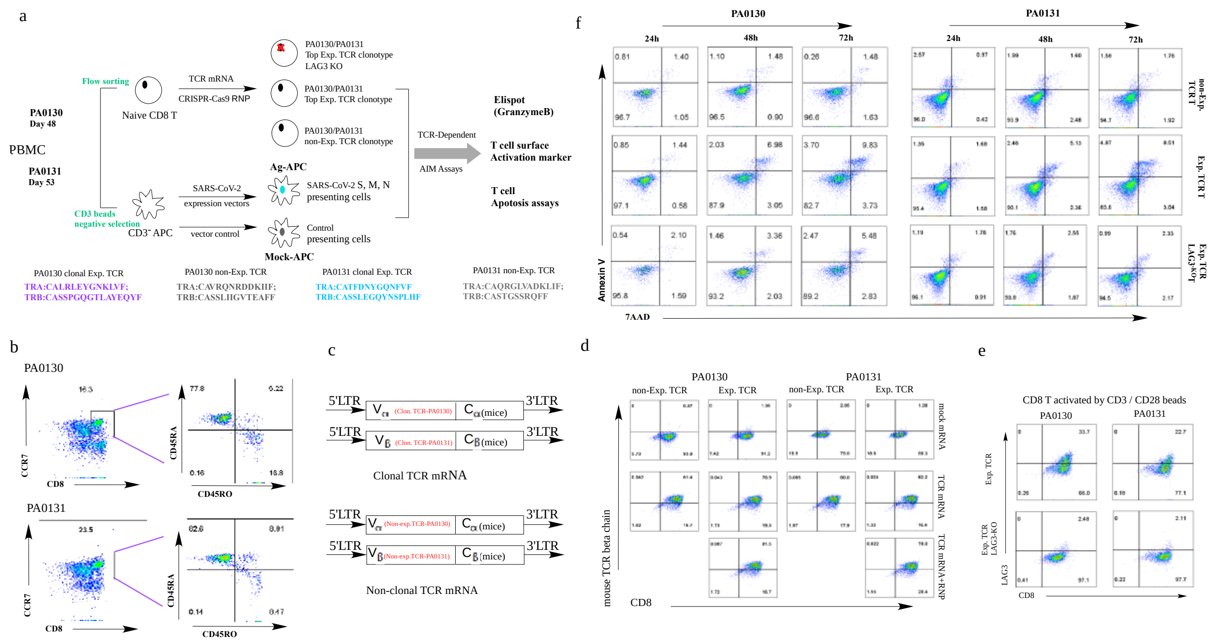

Fig. S4 Dominant clones of T cells identified during infection are SARS-CoV-2 specific and construction of SARS-CoV-2 TCR-T cell and LAG3 knock-out detection. (a) Overview of SARS-CoV-2 TCR-T cell construction and functional analyses. The top 1 clonotype in PA0130 and 0131 were selected as clonal TCR, and TCR sequences from one cell per clone in each patient were selected as non-expanded TCR. (b) Flow cytometry cell sorting of naïve CD8+ T cells from PBMC of patient 0130 and 0131 at timepoint 2. (c) Schematic illustration of RNA expression vectors encoding clonal expanded and non-expanded TCR expression cassette. (d) Flow cytometric analysis of TCR transfected CD8+ T cells, transfection efficacy was indicated by mouse TCR beta chain expression. (e) LAG3 expression level was detected by FACS 48h after TCR-T cells activation by incubation with CD3/CD28 microbeads. (f) Flow cytometric plots showing percentage of apoptotic TCR-T cells after co-culturing with Ag-loaded APCs for the indicated duration.

Table S1. Patient sampling time and dataset information

Patient/Health Donor	Sample Type	Sampling Time (post-symptom onset)			Single Cell Data Type
**PA0130**	PBMC	Day 6 (Time 1)	Day 48 (Time 2)	Day 107 (Time 3)	Single-cell Gene Expression +TCR Seq +BCR Seq
**PA0131**		Day 7 (Time 1)	Day 53 (Time 2)	Day 109 (Time 3)	
**PA0132**		Day 4 (Time 1)		Day 127 (Time 3)	
**PA0133**		Day 5 (Time 1)		Day 125 (Time 3)	
**PA0134**		Day 4 (Time 1)		Day 110 (Time 3)	

Table S2. Clinical characteristics of COVID-19 patients PA0130-0134 and 2 health donors

	PA0130	PA0131	PA0132	PA0133	PA0134	Health donor 1, 2
**Age (Y)**	29	32	62	56	34	33, 60
**Gender**	male	male	female	female	male	M, F
**First symptom**	Fever+ cough	Fever+ cough	Fever	Fever+cough	Fever	N/A
**SARS-CoV-2**	+	+	+	+	+	-
**Influenza A virus**	-	-	-	-	-	-
**Influenza B virus**	-	-	-	-	-	-
**Respiratory syncytial virus**	-	-	-	-	-	-
**Adenovirus**	-	-	-	-	-	-
**CT finding**	Unilateral pneumonia	Unilateral pneumonia	Unilateral pneumonia	Unilateral pneumonia	Unilateral pneumonia	N/A
**Severity**	moderate	moderate	severe	severe	moderate	N/A
**Moxifloxacin**	+	+	-	-	-	N/A
**Lopinavir /Ritonavir**	+	+	+	+	+	N/A
**Acetylcysteine**	-	+	+	+	+	N/A
**Prednisone**	-	+	+	+	+	N/A
**Hospitalization days**	22	14	32	16	13	N/A
**virus clearance days**	21	13	29	14	17	N/A
**Data set**	Single cell Seq, TCR, BCR	Single cell Seq, TCR, BCR	Single cell Seq, TCR, BCR	Single cell Seq, TCR, BCR	Single cell Seq, TCR, BCR	Single cell Seq, TCR, BCR
**Sampling time(post symptom)**	Day 6, 48, 107	Day 7, 53, 109	Day 4, 127	Day 5， 125	Day 4, 110	N/A

Table S3. The top 20 differentially expressed markers of each cluster

Gene	Cluster	avg_logFC	P-value	Adjusted p-value
GNLY	0	2.091	0	0
GZMH	0	1.912	0	0
GZMB	0	1.753	0	0
NKG7	0	1.748	0	0
FGFBP2	0	1.740	0	0
PRF1	0	1.499	0	0
CST7	0	1.395	0	0
KLRD1	0	1.335	0	0
CX3CR1	0	1.318	0	0
CCL5	0	1.310	0	0
PLEK	0	1.250	0	0
GZMA	0	1.213	0	0
LGALS1	0	1.180	0	0
ADGRG1	0	1.178	0	0
SPON2	0	1.101	0	0
EFHD2	0	1.092	0	0
CTSW	0	1.052	0	0
PRSS23	0	1.010	0	0
FCRL6	0	0.996	0	0
LAIR2	0	0.994	0	0
LINC02446	1	1.318	0	0
CD8B	1	0.805	0	0
NELL2	1	0.765	0	0
CCR7	1	0.725	0	0
ACTN1	1	0.685	0	0
LEF1	1	0.651	0	0
LRRN3	1	0.583	0	0
AIF1	1	0.577	0	0
NUCB2	1	0.566	0	0
TCF7	1	0.545	0	0
NT5E	1	0.525	0	0
CARS	1	0.504	0	0
CD7	1	0.501	0	0
RGS10	1	0.479	0	0
EEF1B2	1	0.474	0	0

NOSIP	1	0.473	0	0
RPS5	1	0.471	0	0
TRABD2A	1	0.466	0	0
OXNAD1	1	0.456	2.75E-257	5.36E-253
ABLIM1	1	0.471	5.33E-254	1.04E-249
LTB	2	0.898	0	0
AQP3	2	0.766	0	0
IL7R	2	0.669	0	0
TRADD	2	0.641	0	0
CD4	2	0.629	0	0
CD82	2	0.600	0	0
FXYD5	2	0.533	0	0
VIM	2	0.524	0	0
CD40LG	2	0.474	9.09E-306	1.77E-301
GPR183	2	0.498	1.60E-296	3.12E-292
PLP2	2	0.608	1.42E-280	2.78E-276
RNASET2	2	0.539	1.07E-266	2.09E-262
S100A11	2	0.470	3.96E-246	7.73E-242
GSTK1	2	0.451	6.98E-238	1.36E-233
TRAT1	2	0.486	3.45E-213	6.73E-209
MAL	2	0.500	8.25E-199	1.61E-194
BIRC3	2	0.456	5.94E-189	1.16E-184
TNFSF10	2	0.462	1.77E-158	3.45E-154
LIMS1	2	0.406	1.82E-148	3.56E-144
GATA3	2	0.422	6.96E-129	1.36E-124
CD4	3	0.743	0	0
TCF7	3	0.677	0	0
CCR7	3	0.650	0	0
LEF1	3	0.637	0	0
SELL	3	0.593	0	0
RPS13	3	0.426	0	0
RPS3A	3	0.425	0	0
RPL9	3	0.399	0	0
LTB	3	0.479	1.29E-305	2.51E-301
LDHB	3	0.412	4.39E-271	8.57E-267
MAL	3	0.557	1.12E-266	2.19E-262
NOSIP	3	0.554	1.17E-266	2.28E-262
IL6ST	3	0.506	3.17E-201	6.19E-197
TRABD2A	3	0.486	1.90E-178	3.70E-174

EEF1G	3	0.404	4.68E-177	9.14E-173
CAMK4	3	0.473	1.02E-176	2.00E-172
RCAN3	3	0.419	1.63E-138	3.19E-134
ABLIM1	3	0.401	4.03E-127	7.87E-123
LINC00861	3	0.408	6.24E-120	1.22E-115
AC243960.1	3	0.403	5.39E-113	1.05E-108
GZMK	4	1.751	0	0
CCL5	4	0.837	0	0
GZMA	4	0.813	2.11E-238	4.12E-234
CD74	4	0.747	1.47E-207	2.86E-203
CST7	4	0.620	9.82E-200	1.92E-195
CMC1	4	0.756	3.08E-140	6.01E-136
HLA-DPA1	4	0.556	2.15E-134	4.20E-130
GZMM	4	0.490	5.41E-102	1.06E-97
DUSP2	4	0.702	4.47E-97	8.74E-93
HLA-DRB1	4	0.487	1.25E-95	2.43E-91
ISG20	4	0.556	6.38E-95	1.24E-90
IKZF3	4	0.537	1.14E-93	2.23E-89
LYAR	4	0.460	2.10E-87	4.10E-83
KLRG1	4	0.506	6.89E-83	1.34E-78
MT2A	4	0.608	2.35E-79	4.58E-75
CCL4	4	0.546	1.43E-73	2.80E-69
BST2	4	0.478	1.54E-69	3.00E-65
ISG15	4	0.604	2.79E-69	5.45E-65
IFI6	4	0.555	1.67E-66	3.26E-62
MX1	4	0.542	1.57E-61	3.07E-57
FOS	5	2.383	0	0
JUNB	5	1.904	0	0
CD69	5	1.882	0	0
DUSP1	5	1.852	0	0
JUN	5	1.787	0	0
NR4A2	5	1.677	0	0
ZFP36	5	1.628	0	0
GADD45B	5	1.566	0	0
BTG2	5	1.560	0	0
FOSB	5	1.538	0	0
PPP1R15A	5	1.472	0	0
NFKBIA	5	1.460	0	0
SLC2A3	5	1.418	0	0

TSC22D3	5	1.324	0	0
YPEL5	5	1.179	0	0
DDIT4	5	1.107	0	0
CSRNP1	5	1.079	0	0
DNAJB1	5	1.057	2.97E-305	5.81E-301
KLF6	5	1.125	7.72E-218	1.51E-213
RGCC	5	1.059	7.66E-217	1.50E-212
RPS10	6	1.418	0	0
DUSP2	6	1.294	0	0
MT-ATP6	6	1.278	0	0
CREM	6	1.228	0	0
FGFBP2	6	1.094	0	0
DDIT4	6	1.066	0	0
SRGN	6	1.057	0	0
CXCR4	6	1.026	0	0
VAMP2	6	0.967	0	0
NKG7	6	0.922	0	0
SLC7A5	6	0.880	0	0
GABARAPL1	6	0.874	0	0
MT-ND4	6	0.846	0	0
CCL5	6	0.827	2.21E-293	4.32E-289
NR4A2	6	0.888	8.01E-263	1.56E-258
SLA2	6	0.830	1.91E-235	3.73E-231
LDHA	6	0.851	3.03E-228	5.91E-224
IRF1	6	0.833	1.12E-215	2.19E-211
FAM177A1	6	0.913	6.70E-211	1.31E-206
TRBV28	6	1.057	3.48E-134	6.79E-130
RPS10	7	1.845	0	0
MT-ATP6	7	1.194	0	0
MT-ND4	7	0.919	0	0
SARAF	7	0.726	1.13E-307	2.21E-303
RPS3A	7	0.537	2.63E-302	5.14E-298
RPL5	7	0.534	4.49E-299	8.77E-295
RPL36A	7	0.735	1.08E-269	2.10E-265
MT-CYB	7	0.614	1.65E-253	3.23E-249
LEPROTL1	7	0.844	6.32E-245	1.23E-240
ATP5PO	7	0.731	1.66E-244	3.23E-240
NDUFB8	7	0.578	5.75E-236	1.12E-231
CCR7	7	0.821	4.29E-234	8.38E-230

RPS4Y1	7	0.680	3.79E-219	7.39E-215
CD55	7	0.716	3.51E-184	6.86E-180
SOCS1	7	0.591	2.99E-169	5.84E-165
NDUFA11	7	0.589	1.53E-160	2.98E-156
PIK3IP1	7	0.615	2.97E-143	5.81E-139
VAMP2	7	0.585	1.68E-137	3.28E-133
LINC02446	7	0.605	2.12E-105	4.15E-101
PASK	7	0.571	1.57E-95	3.07E-91
PPBP	8	2.501	0	0
NRGN	8	1.689	0	0
TUBB1	8	1.687	0	0
PF4	8	1.637	0	0
CAVIN2	8	1.596	0	0
HIST1H2AC	8	1.519	0	0
GNG11	8	1.442	0	0
SPARC	8	1.441	0	0
CLU	8	1.438	0	0
RGS18	8	1.360	0	0
MYL9	8	1.213	0	0
TSC22D1	8	1.202	0	0
GP9	8	1.145	0	0
MPIG6B	8	1.124	0	0
HIST1H3H	8	1.077	0	0
MAP3K7CL	8	1.019	0	0
MMD	8	0.904	0	0
F13A1	8	0.897	0	0
TREML1	8	0.880	0	0
PGRMC1	8	0.831	4.57E-242	8.92E-238
KLRB1	9	2.046	0	0
TRAV1-2	9	1.658	0	0
GZMK	9	1.533	0	0
NCR3	9	1.485	0	0
TRBV6-4	9	1.209	0	0
SLC4A10	9	0.986	0	0
CEBPD	9	0.772	0	0
RORC	9	0.642	0	0
CXCR6	9	0.595	0	0
ZBTB16	9	0.570	0	0
KLRG1	9	1.079	5.83E-294	1.14E-289

IL7R	9	1.015	2.35E-217	4.58E-213
GZMA	9	0.659	1.42E-163	2.78E-159
DPP4	9	0.567	4.77E-142	9.31E-138
ERN1	9	0.615	1.17E-114	2.28E-110
ALOX5AP	9	0.664	3.11E-114	6.07E-110
AQP3	9	0.622	1.39E-106	2.71E-102
GBP5	9	0.644	4.88E-106	9.53E-102
GPR65	9	0.675	8.18E-102	1.60E-97
JAML	9	0.557	1.07E-87	2.08E-83
TYROBP	10	1.128	0	0
KLRF1	10	0.735	0	0
TRDC	10	0.595	0	0
SH2D1B	10	0.462	0	0
FCGR3A	10	0.817	1.39E-156	2.72E-152
SPON2	10	0.958	2.81E-118	5.49E-114
GNLY	10	1.065	7.96E-106	1.55E-101
FCER1G	10	0.658	1.46E-105	2.85E-101
CLIC3	10	0.630	4.48E-99	8.74E-95
KLRD1	10	0.558	8.56E-93	1.67E-88
HOPX	10	0.514	2.19E-90	4.28E-86
TTC38	10	0.396	2.71E-81	5.28E-77
GZMB	10	0.666	2.14E-75	4.17E-71
PRF1	10	0.770	4.49E-72	8.77E-68
IL2RB	10	0.464	1.11E-69	2.17E-65
CTSW	10	0.529	1.10E-54	2.14E-50
FGFBP2	10	0.393	1.29E-53	2.51E-49
CCL4	10	0.415	8.38E-52	1.64E-47
CD247	10	0.473	1.59E-49	3.10E-45
CST7	10	0.394	6.59E-38	1.29E-33
S100A8	11	4.411	0	0
S100A9	11	3.903	0	0
LYZ	11	3.644	0	0
CST3	11	3.290	0	0
TYROBP	11	2.945	0	0
FCN1	11	2.783	0	0
FCER1G	11	2.594	0	0
SERPINA1	11	2.286	0	0
AIF1	11	2.133	0	0
HLA-DRA	11	2.120	0	0

CCL3	11	2.115	0	0
IL1B	11	2.111	0	0
LST1	11	2.089	0	0
S100A12	11	1.975	0	0
MNDA	11	1.938	0	0
GRN	11	1.897	0	0
SPI1	11	1.871	0	0
CTSS	11	1.857	0	0
IFI27	11	2.696	1.43E-239	2.80E-235
IFITM3	11	2.402	2.94E-152	5.74E-148
FOXP3	12	1.608	0	0
IL2RA	12	0.981	0	0
RTKN2	12	0.949	0	0
IKZF2	12	0.637	2.40E-149	4.69E-145
CTLA4	12	0.643	7.62E-142	1.49E-137
CD4	12	0.679	1.91E-127	3.74E-123
STAM	12	0.656	5.31E-118	1.04E-113
TIGIT	12	0.954	6.99E-111	1.37E-106
IL32	12	0.773	1.08E-75	2.10E-71
LGALS3	12	0.831	2.42E-74	4.72E-70
SHMT2	12	0.661	1.42E-59	2.77E-55
BIRC3	12	0.707	1.32E-57	2.57E-53
GBP5	12	0.830	2.99E-54	5.85E-50
SELL	12	0.686	1.26E-50	2.46E-46
ISG20	12	0.698	1.30E-46	2.53E-42
IL10RA	12	0.685	6.55E-45	1.28E-40
OAS1	12	0.680	9.40E-37	1.83E-32
MT2A	12	0.859	7.98E-21	1.56E-16
MX1	12	0.657	8.07E-20	1.57E-15
ISG15	12	0.687	7.66E-16	1.49E-11
TYMS	13	1.157	0	0
MT1E	13	1.023	0	0
CD38	13	0.962	0	0
HLA-DRA	13	0.899	4.07E-175	7.94E-171
GZMK	13	1.092	4.87E-158	9.50E-154
PFN1	13	0.899	2.27E-152	4.43E-148
GAPDH	13	1.214	2.09E-150	4.07E-146
ACTG1	13	1.116	2.41E-143	4.71E-139
COTL1	13	1.198	8.11E-130	1.58E-125

CD74	13	1.056	6.80E-111	1.33E-106
STMN1	13	1.701	1.18E-109	2.30E-105
H2AFV	13	0.922	1.12E-102	2.18E-98
MT2A	13	1.010	5.65E-101	1.10E-96
GZMA	13	1.042	4.29E-81	8.37E-77
HMGB2	13	1.329	5.63E-80	1.10E-75
IFI27	13	1.474	1.29E-65	2.51E-61
HMGN2	13	1.052	5.18E-58	1.01E-53
TUBB	13	1.022	1.13E-31	2.20E-27
TUBA1B	13	1.362	4.21E-17	8.22E-13
HIST1H4C	13	1.017	1.75E-16	3.41E-12
KLRB1	14	2.088	0	0
AC020916.1	14	1.619	0	0
TRAV1-2	14	1.337	0	0
NCR3	14	1.341	1.54E-285	3.00E-281
TRBV6-4	14	1.306	1.60E-284	3.13E-280
GZMK	14	1.115	4.91E-228	9.58E-224
DUSP1	14	2.123	4.88E-195	9.52E-191
DUSP2	14	1.993	1.52E-191	2.96E-187
NFKBIA	14	1.872	9.54E-169	1.86E-164
CD69	14	1.570	7.55E-164	1.47E-159
TNFAIP3	14	1.518	8.90E-157	1.74E-152
ZFP36	14	1.939	3.80E-154	7.42E-150
CXCR4	14	1.256	1.03E-141	2.02E-137
BHLHE40	14	0.992	1.75E-120	3.41E-116
JUNB	14	1.140	2.80E-117	5.46E-113
ZFP36L2	14	0.946	2.99E-105	5.84E-101
PPP1R15A	14	1.056	4.14E-95	8.09E-91
FOS	14	1.332	1.19E-85	2.31E-81
JUN	14	1.230	9.42E-67	1.84E-62
IER2	14	1.009	4.90E-62	9.57E-58
MALAT1	15	1.461	2.01E-134	3.93E-130
PTPRC	15	1.707	3.97E-123	7.75E-119
CD4	15	1.433	7.74E-73	1.51E-68
IL7R	15	1.625	1.90E-54	3.71E-50
RNF213	15	1.441	1.54E-53	3.02E-49
IFI44L	15	1.412	5.00E-43	9.76E-39
NEAT1	15	1.501	7.73E-36	1.51E-31
ITGB1	15	1.612	6.02E-32	1.17E-27

KLF6	15	1.222	1.47E-30	2.88E-26
HSPA5	15	1.269	2.05E-27	4.01E-23
TNFSF10	15	1.415	7.19E-26	1.40E-21
DDX17	15	1.258	1.54E-25	3.01E-21
MACF1	15	1.410	6.64E-25	1.30E-20
RSAD2	15	1.267	1.33E-23	2.59E-19
SMCHD1	15	1.291	2.72E-23	5.31E-19
SYNE2	15	1.279	2.04E-21	3.98E-17
APOL6	15	1.277	2.60E-21	5.07E-17
LNPEP	15	1.187	1.04E-20	2.03E-16
SLC2A3	15	1.404	1.37E-14	2.67E-10
CD69	15	1.371	8.90E-05	1

Table S4 Differentially expressed genes across three time points for clonal CD8+ T cells of 5 patients

Gene	Cluster	avg_logFC	P-value	Adjusted p-value
CXCR4	T1	-1.404	9.95E-129	1.94E-124
DUSP2	T1	-1.371	5.04E-91	9.85E-87
ZFP36	T1	-1.115	9.21E-70	1.80E-65
NFKBIA	T1	-1.043	1.60E-41	3.12E-37
FOS	T1	-1.056	1.45E-07	0.002827236
IFI6	T2	-1.242	1.88E-37	3.67E-33
ISG15	T2	-1.117	7.72E-37	1.51E-32
MX1	T2	-1.103	3.24E-35	6.33E-31
CXCR4	T3	1.348	2.95E-145	5.75E-141
DUSP2	T3	1.412	1.78E-113	3.47E-109
ZFP36	T3	1.231	2.30E-93	4.48E-89
NFKBIA	T3	1.288	4.50E-79	8.79E-75
RPS10	T3	1.049	4.65E-52	9.08E-48

Table S5 The CDR3 and consensus sequences of TRA and TRB for 4 T cell clones of PA0130 and PA0131 used in TCR-dependent AIM assays

Patient	CDR3	Gene	Consensus sequence
PA0130 Clonal Exp. TCR	TRA:CALRLEYGNKLVF	TRAV19	MLTASLLRAVIASICVVSSMAQKVTQAQTEISVVEKEDVTLDCVYE TRDTTYYLFWYKQPPSGELVFLIRRNSFDEQNEISGRYSWNFQKSTS SFNFTITASQVVDSAVYFCALRLEYGNKLVFGAGTILRVKSYIQNPD PAVYQLRD
	TRB:CASSPGQGTLAYE QYF	TRBV4-1	MGCRLLCCAVLCLLGAVPIDTEVTQTPKHLVMGMTNKKSLKCEQH MGHRAMYWYKQKAKKPPELMFVYSYEKLSINESVPSRFSPECPNSS LLNLHLHALQPEDSALYLCASSPGQGTLAYEQYFGPGTRLTVTEDL KNVFPPEVAVFEPS
PA0130 non-Exp. TCR	TRA:CAVRQNRDDKIIF	TRAV21	METLLGLLILWLQLQWVSSKQEVTQIPAALSVPEGENLVLNCSFTDS AIYNLQWFRQDPGKGLTSLLLIQSSQREQTSGRLNASLDKSSGRSTL YIAASQPGDSATYLCAVRQNRDDKIIFGKGTRLHILPNIQNPDPAVY QLRD
	TRB:CASSLIIGVTEAFF	TRBV11-2	MGTRLLCWAALCLLGAELTEAGVAQSPRYKIIEKRQSVAFWCNPIS GHATLYWYQQILGQGPKLLIQFQNNGVVDDSQLPKDRFSAERLKG VDSTLKIQPAKLEDSAVYLCASSLIIGVTEAFFGQGTRLTVVEDLNK VFPPEVAVFEPS
PA0131 Clonal Exp. TCR	TRA:CATFDNYGQNFVF	TRAV17	METLLGVSLVILWLQLARVNSQQGEEDPQALSIQEGENATMNCSYK TSINNLQWYRQNSGRGLVHLILIRSNEREKHSGRLRVTLDTSKKSSS LLITASRAADTASYFCATFDNYGQNFVFGPGTRLSVLPYIQNPDPAV YQLRD
	TRB:CASSLEGQYNSPL HF	TRBV28	MGIRLLCRVAFCFLAVGLVDVKVTQSSRYLVKRTGEKVFLECVQD MDHENMFWYRQDPGLGLRLIYFSYDVKMKEKGDIPEGYSVSREKK ERFSLILESASTNQTSMYLCASSLEGQYNSPLHFGNGTRLTVTEDLN KVFPPEVAVFEPS
PA0131 non-Exp. TCR	TRA:CAQRGLVADKLIF	TRAV13-1	MTSIRAVFIFLWLQLDLVNGENVEQHPSTLSVQEGDSAVIKCTYSDS ASNYFPWYKQELGKRPQLIIDIRSNVGEKKDQRIAVTLNKTAKHFSL HITETQPEDSAVYFCAQRGLVADKLIFGTGTRLQVFPNIQNPDPAVY QLRD
	TRB:CASTGSSRQFF	TRBV7-2	MGTRLLFWVAFCLLGADHTGAGVSQSPSNKVTEKGKDVELRCDPI SGHTALYWYRQSLGQGLEFLIYFQGNSAPDKSGLPSDRFSAERTGG SVSTLTIQRTQQEDSAVYLCASTGSSRQFFGPGTRLTVLEDLKNVFP PEVAVFEPS

Table S6 HLA information of PA0130 and PA0131

Patient	A	B	C	DRB1	DQB1	DPB1	DQA1	DPA1	DRB345
PA0130	02:01<br>02:07	13:01<br>51:02	01:02<br>08:01	09:01<br>11:01	03:01<br>03:03	02:01<br>05:01	03:02<br>05:05	01:03<br>02:02	DRB3*02:02<br>DRB4*01:03
PA0131	02:03<br>11:01	40:01<br>40:01	03:04<br>07:02	04:04<br>08:03	03:02<br>06:01	02:01<br>05:01	01:03<br>03:01	02:02<br>02:02	DRB4*01:03<br>NP

Table S7 Single-cell mapping statistics of samples PA0130-PA0134 and Health Donor 01-02

	PA0130 (Time 1)	PA0130 (Time2)	PA0130 (Time 3)	PA0131 (Time 1)	PA0131 (Time 2)	PA0131 (Time 3)	PA0132 (Time 1)	PA0132 (Time 3)	PA0133 (Time 1)	PA0133 (Time 3)	PA0134 (Time 1)	PA0134 (Time 3)	Health donor 1	Health donor 2
Gene expression														
Number of reads	335879426	329671149	390207199	365545825	319987847	361840383	367573942	384132485	370964516	407963874	325924817	285802571	399179914	401162321
Number of cells	6522	7865	7794	6003	8376	9705	8716	4989	8142	5625	6115	7530	7282	6054
Median Genes per Cell	1438	1306	1214	1512	1292	1158	1309	1476	1398	1387	1338	1072	1461	1353
Total Genes Detected	19570	19364	19035	19315	19356	19433	19554	18903	19621	19373	18872	18790	19620	19607
Reads Mapped to Genome	0.923	0.931	0.948	0.917	0.906	0.945	0.906	0.931	0.917	0.938	0.93	0.814	0.926	0.916
TCR														
Number of Read Pairs	32390674	33436346	38583709	30104428	46339304	39274606	32089356	25224560	29538354	25902285	34949106	33790127	35230205	30342810
Number of Cells	2533	3901	3480	2386	3206	3533	2974	2335	3134	2418	2881	3912	4164	1834
Number of Cells With Productive V-J Spanning Pair	2167	3292	2827	2061	2887	3042	2475	2004	2641	2003	2245	2494	3478	1322
Reads Mapped to Any V(D)J Gene	0.803	0.806	0.819	0.811	0.797	0.772	0.81	0.791	0.831	0.816	0.811	0.795	0.832	0.764
Number of Clonotypes	1941	3073	2658	1591	2531	2844	2498	2160	2356	2064	1982	2807	4030	1734
BCR														
Number of Read Pairs	26536828	25838369	40326568	28711299	28611995	37807031	47949217	25348768	26900813	26747391	29765007	27760586	26954522	27307929
Number of Cells	1475	1307	1905	629	867	1530	1248	1067	433	482	761	555	764	1126
Number of Cells With Productive V-J Spanning Pair	1363	1239	1761	573	832	1394	1064	984	359	455	693	457	699	1052
Reads Mapped to Any IGH/IGK/IGL Gene	0.83	0.756	0.67	0.801	0.711	0.598	0.809	0.768	0.622	0.666	0.769	0.625	0.657	0.709
Number of Clonotypes	1444	1277	1752	579	864	1277	1025	988	393	448	699	525	749	1103
